# Whatever happened to China’s neglected tropical diseases?

**DOI:** 10.1186/s40249-019-0598-5

**Published:** 2019-10-02

**Authors:** Peter J. Hotez

**Affiliations:** 1 Texas Children’s Hospital Center for Vaccine Development, Departments of Pediatrics and Molecular Virology & Microbiology, National School of Tropical Medicine, Baylor College of Medicine, Houston, TX USA; 2 Hagler Institute for Advanced Studies at Texas A&M University, College Station, TX USA; 3 Department of Biology, Baylor University, Waco, TX USA; 4 James A Baker III Institute of Public Policy, Rice University, Houston, TX USA; 5 Scowcroft Institute of International Affairs, Texas A&M University, College Station, TX USA

**Keywords:** Control, Elimination, Neglected tropical diseases, Parasitic infections, People’s Republic of China, Poverty reduction

## Abstract

Before the founding of the People’s Republic of China 70 years ago, both extreme poverty and parasitic infections and other neglected tropical diseases were highly prevalent. Owing to social development, particularly economic reforms since the 1980s, poverty has since been dramatically reduced, and China became increasingly urbanized and industrialized. In parallel, China’s economic transformation translated into similar and remarkable reductions in neglected tropical diseases. Qian and colleagues report in their review published in *Infectious Diseases of Poverty*, the elimination or near elimination as a public health problem of lymphatic filariasis, trachoma, soil-transmitted helminth infections, schistosomiasis and other neglected tropical diseases. Of note, neglected tropical disease control and poverty reduction each appear to reinforce the other. China’s formula for success in parasitic and neglected tropical disease control might translate to other parts of the world, such as in sub-Saharan Africa through China’s new Belt and Road Initiative.

## Multilingual abstracts

Please see Additional file 1 for translations of the abstract into the five official working languages of the United Nations.

The People’s Republic of China has just marked the 70th anniversary of founding, and there is much to celebrate in terms of China’s reductions in both poverty and poverty-related neglected tropical diseases. Prior to 1949, many disparaged China as the “Sick Man of Asia” [1], referring to the exploitation by foreign powers and economic downturns that trapped hundreds of millions of Chinese in the most wrenching destitution the world had known at that time [1]. Not surprisingly, China’s deprivations went hand-in-hand with extraordinary rates of parasitic infections and other neglected tropical diseases. Indeed, W.A. Scott, a Canadian working and living in Shanghai before 1949 graphically described, “children covered with horrible sores upon which flies feasted ( …) children having a bowel movement, which, after much strain, would only eject tapeworms” [2].

As late as the 1980s, China’s own national surveys revealed that most of its population, especially in rural areas, was still infected with intestinal worms, schistosomes, food-borne trematodes or filarial nematodes, while malaria, tuberculosis and other poverty-related conditions were also widespread [3]. China was a polyparasitized nation with dire economic consequences – worm infections and other neglected tropical diseases arise in poverty, but also they can accelerate poverty due to their chronic and debilitating effects [4, 5].

And then, an economic miracle occurred. Initially, under the leadership of Deng Xiaoping, economic reforms began in the 1980s and extended into the twenty-first century, lifting more than 800 million Chinese out of extreme poverty [6]. Eventually, the number of people living below the World Bank poverty level of US$ 1.90 per day fell below 750 million, with much of those gains resulting from poverty reductions in China. Over this period, agricultural gains increased and malnutrition rates fell [6]. China’s population became increasingly urbanized and industrialized [6], especially in the Eastern part of the country.

As Qian et al. report in a major review published in *Infectious Diseases of Poverty*, China’s economic gains ran parallel with similar and remarkable reductions in neglected tropical diseases [7]. Both lymphatic filariasis and trachoma were eliminated as a public health problem, while the national prevalence of soil-transmitted helminth infections fell from over 50% (as determined from the results of China’s first parasite survey between 1988 and 1992) to just under 5% in 2014–2015 [7]. Similarly, the total number of Chinese living with schistosomiasis decreased from over 11 million in the 1950s, to under 40 000 in 2017 [7]. Dramatic reductions also occurred for cestode and food-borne trematode infections, as well as leishmaniasis, leprosy and rabies [7].

There is little doubt that mass drug administration together with improvements in water, sanitation and hygiene were instrumental in reducing China’s disease burden from parasitic worm infections. In the case of Asian schistosomiasis, a zoonosis from water buffalo and pigs, removal of animal reservoirs and tractor mechanization also had an important public health impact. Essential to disease control were the stalwart efforts of China’s parasitology research community, including the National Institute of Parasitic Diseases of the Chinese Center for Disease Control and Prevention.

A key question for China is “which came first”? Did parasite control improve the health of a nation and create a tipping point that promoted economic development, or did parasites decline due to national economic reforms, industrialization and urbanization? There remains an urgency for social scientists to explore these relationships in more detail, but it is likely that neglected tropical disease control and poverty reduction each reinforce the other (Fig. 1).

**Fig. 1:**
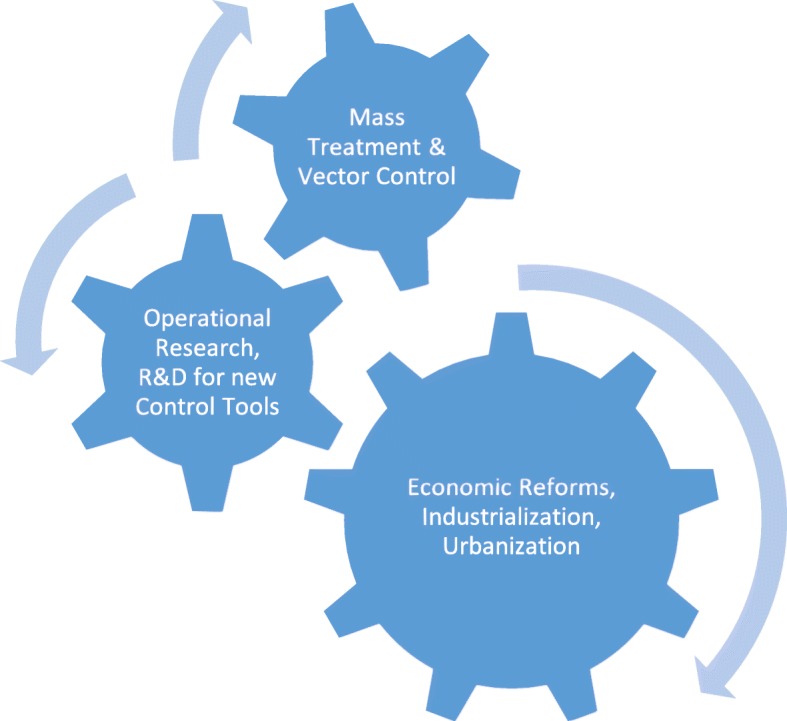
The three pillars of parasite and neglected tropical disease elimination

Therefore, economic reforms, urbanization and industrialization accelerated the decline in China’s neglected tropical disease burden, just as it did in the southern parts of the United States of America during the first half of the twentieth century, and in Japan and Korea during the 1950s, 1960s and 1970s [8–10]. These observations may also help to explain why both extreme poverty and parasitic infections remain significant in China’s poorer southwestern provinces.

Another important and remaining unanswered question is whether China’s formula for success in parasitic and neglected tropical disease control might translate to other parts of the world, such as in sub-Saharan Africa where these diseases remain widespread. In particular, can the control of poverty-related parasitic and neglected tropical diseases succeed in the absence of aggressive economic reforms? We still do not have strong evidence that mass treatment with existing drugs can eliminate neglected tropical diseases in African or related settings of high parasite prevalence and intensity, or whether additional technologies, including improved drugs and new vaccines, will become essential.

Finally, China’s new Belt and Road Initiative will create unprecedented links between sub-Saharan Africa and China, and potentially incentivize China’s parasitic disease experts to reproduce their past successes on the African continent. On the other hand, an unprecedented level of human movements between Africa and East Asia will also create the potential for introducing neglected tropical diseases into China or promoting re-emergence [11]. There are concerns that malaria due to *Plasmodium falciparum*, yellow fever and other arbovirus infections, or even haemorrhagic fever viruses could one day gain a foothold in China. A new level of disease surveillance and patient treatment for tropical diseases may become necessary. The coming decade will almost certainly create remarkable times for both China and Africa’s parasitic and neglected tropical disease control and elimination, thereby contributing to achieving several of the Sustainable Development Goals.

## Supplementary information

**Additional file 1:** Multilingual abstracts in the five official working languages of the United Nations. (PDF 252 kb)

## Data Availability

Not applicable.

## References

[1] Hotez PJ (2002). China’s hookworms. China Q.

[2] Scott WA (1966). China revisited by an old China hand. Eastern Horizon.

[3] Hotez PJ, Zheng F, Long-qi X, Ming-gang C, Shu-hua X, Shu-xian L (1997). Emerging and reemerging helminthiases and the public health of China. Emerg Infect Dis.

[4] Lenk EJ, Redekop WK, Luyendijk M, Rijnsburger AJ, Severens JL (2016). Productivity loss related to neglected tropical diseases eligible for preventive chemotherapy: a systematic literature review. PLoS Negl Trop Dis.

[5] Blouin B, Casapia M, Joseph L, Gyorkos TW (2018). A longitudinal cohort study of soil-transmitted helminth infections during the second year of life and associations with reduced long-term cognitive and verbal abilities. PLoS Negl Trop Dis.

[6] Huang Z, Lahiri T. China’s path out of poverty can never be repeated at scale by a country again. Quartz September. 2017;20 https://qz.com/1082231/chinas-path-out-of-poverty-can-never-be-repeated-at-scale-by-any-other-country/. Accessed 13 Sept 2019.

[7] Qian MB, Chen J, Bergquist R, Li ZJ, Li SZ, Xiao N, et al. Neglected tropical diseases in the People’s republic of China: progress towards elimination. Infect Dis Poverty. 10.1186/s40249-019-0599-4. 10.1186/s40249-019-0599-4 PMC6775666 31578147

[8] Humphreys M (2009). How four once common diseases were eliminated from the Americ South. Health Aff (Millwood).

[9] Hong ST, Chai JY, Choi MH, Huh S, Rim HJ, Lee SH (2006). A successful experience of soil-transmitted helminth control in the Republic of Korea. Korean J Parasitol.

[10] Kasai T, Nakatani H, Takeuchi T, Crump A (2007). Research and control of parasitic diseases in Japan: current position and future perspectives. Trends Parasitol.

[11] Wang L, Zou Y, Zhu X, Bottazzi ME, Hotez PJ, Zhan B (2019). China’s shifting neglected parasitic infections in an era of economic reform, urbanization, disease control, and the belt and road initiative. PLoS Negl Trop Dis.

