# Supplementary material for: Whatever happened to China’s neglected tropical diseases?
Source: Infect Dis Poverty. 2019 Oct 2;8:85. doi: 10.1186/s40249-019-0598-5 (PMC6775646; doi:10.1186/s40249-019-0598-5)
Supplement: Supplementary file 1 — Additional file 1: Multilingual abstracts in the five official working languages of the United Nations. (PDF 252 kb) [file 40249_2019_598_MOESM1_ESM.pdf]

Translation of the abstract into the five official working languages of the United Nations

ترى ما هي أخبار الأمراض المدارية المهملة في الصين؟

بيتر ج. هوتز

الملخص

قبل تأسيس جمهورية الصين الشعبية منذ ٧٠ عامًا كان كلا من الفقر المدقع والعدوي الطفيلية وغيرها من الأمراض المدارية المهملة منتشرة بشكل كبير ولكن بسبب التنمية الاجتماعية ولا سيما الإصلاحات الاقتصادية انخفض الفقر بشكل كبير منذ الثمانينات وأصبحت الصين حضرية وصناعية بشكل متزايد وبالتوازي مع ذلك ترجم التحول الاقتصادي في الصين إلى تخفيضات مماثلة وملحوظة في الأمراض المدارية المهملة ويشير تشيان وآخرون في هذا العدد من مجلة الأمراض المعدية المرتبطة بالفقر إلي القضاء أو قرب القضاء على مرض الفيلاريات اللمفاوية والتراخوما وعدوي الديدان الطفيلية المنقولة بالتربة وداء البلهارسيات وغيرها من الأمراض المدارية المهملة كمشاكل صحية عامة في الصين ويبدو أن السيطرة على الأمراض المدارية المهملة والحد من الفقر يعزز كل منهما الآخر ومن الممكن ان تنتقل وصفة الصين للنجاح في مكافحة الأمراض الطفيلية والمهملة إلى أجزاء أخرى من العالم علي سبيل المثال في جنوب الصحراء الكبرى في أفريقيا من خلال مبادرة الحزام والطريق الجديدة في الصين.

Translated from English version into Arabic by Mohamed Habib

中国 “被忽视的热带病”的防控进展与方向

Peter J Hotez

摘要

在中华人民共和国成立以前，中国极端贫困，寄生虫病和其他“被忽视的热带病”肆虐。中国人民共和国成立以后，随着社会的发展，尤其是改革开放以来，中国逐渐改变了积贫积弱的局面，城镇化和工业化程度显著提高。中国的经济发展也促进了“被忽视的热带病”的防控。在《贫困所致传染病》英文期刊上，Qian 等人报告了防控这些疾病取得的成效，如淋巴丝虫病、沙眼、土源性蠕虫病、血吸虫病和其他“被忽视热带病”已被消除或接近消除。“被忽视热带病”的控制和贫困的消除互为因果。随着一带一路的深入发展，中国在寄生虫病和其他“被忽视热带病”方面防治经验可分享给全球其他地方，如撒哈拉以南非洲的流行区。

Translated from English version into Chinese by Men-Bao Qian

Qu'est-il arrivé aux maladies tropicales négligées de la Chine?

Peter J. Hotez

Resumé

Soixante- dix ans avant la fondation de la République Populaire de Chine, l'extrême pauvreté, les infections parasitaires et autres maladies tropicales négligées étaient très répandues. Grâce au développement social, en particulier les réformes économiques entreprises depuis les années 80, la pauvreté a considérablement diminué et la Chine s'est de plus en plus urbanisée et industrialisée.

Parallèlement, la transformation économique du pays s'est traduite par des réductions similaires et remarquables des maladies tropicales négligées. Les travaux de Qian et collaborateurs publiés dans la revue *Infectious Diseases of Poverty* rapportent l'élimination ou la quasi-élimination comme problème de santé publique de la filariose lymphatique, du trachome, des infections causées par les vers parasitaires transmis par le sol, de la schistosomiase et autres maladies tropicales négligées. Il convient de noter que la lutte contre les maladies tropicales négligées et la réduction de la pauvreté semblent se renforcer mutuellement. La formule adoptée par la Chine pour réussir la lutte contre les maladies tropicales parasitaires et négligées pourrait s'appliquer à d'autres régions du monde, comme l'Afrique subsaharienne dans le cadre de la nouvelle initiative chinoise “Belt and Road”.

Translated from English version into French by Golou L. Bellai

Что случилось с забытыми тропическими болезнями Китая?

Peter J Hotez

Аннотация

70 лет назад, т.е. еще до образования Китайской народной республики намечалась крайняя нищета и были широко распространены паразитические инфекции и другие забытые тропические болезни. В результате социального развития, особенно благодаря экономической реформе с 1980-х годов, число бедных значительно сократилось. Китай уже стал более урбанизированным и индустриализированным. Вместе с тем, экономические преобразования Китая провели к подобному и заметному сокращению забытых тропических болезей. В выпуске «Инфекционные болезни бедности» Цянь и его коллеги заявили об устранении или почти полном устранении лимфатических филяриозов, трахом, инфекций геогельминтов, шистосомозов и других забытых тропических болезней в качестве проблем общественной гигиены. Вроде контроль забытых тропических болезей и снижение уровня бедности усиливают друг друга. Китайский опыт в успешной борьбе с паразитическими и забытыми болезнями может распространяться и в другие регионы мира, например, в страны Африки к югу от Сахары в рамках китайской новой инициативы “Один пояс, один путь”.

Translated from English version into Russian by Yun Li

¿Qué pasó con las enfermedades tropicales desatendidas de China?

Peter J. Hotez

Resumen

Antes de la fundación de la República Popular China hace 70 años, tanto la pobreza extrema como las infecciones parasitarias y otras enfermedades tropicales desatendidas eran muy prevalentes. Debido al desarrollo social, en particular a las reformas económicas desde la década de 1980, la pobreza se ha reducido drásticamente y China se urbanizó e industrializó cada vez más. Durante este mismo periodo, la transformación económica de China se tradujo en reducciones similares y

notables en las enfermedades tropicales desatendidas. En la revisión publicada en Infectious Diseases of Poverty, Qian y colegas informan de la eliminación o casi eliminación como problema de salud pública de la filariasis linfática, el tracoma, las infecciones por geo-helmintos, la eschistosomiasis y otras enfermedades tropicales desatendidas. Cabe destacar que el control de enfermedades tropicales desatendidas y la reducción de la pobreza parecen reforzarse mutuamente. La fórmula de China para el éxito en el control de enfermedades tropicales parasitarias y desatendidas podría transcribirse a otras partes del mundo, como en el África subsahariana a través de la nueva Iniciativa de la Franja y la Ruta de China.

Translated from English version into Spanish Anja Schreier
